# Wheat *Brassinosteroid-Insensitive1 (TaBRI1)* Interacts with Members of *TaSERK* Gene Family and Cause Early Flowering and Seed Yield Enhancement in *Arabidopsis*

**DOI:** 10.1371/journal.pone.0153273

**Published:** 2016-06-20

**Authors:** Akanksha Singh, Priyanka Breja, Jitendra P. Khurana, Paramjit Khurana

**Affiliations:** Department of Plant Molecular Biology, University of Delhi, South Campus, Benito Juarez Road, Dhaula Kuan, New Delhi, 110 021, India; CSIR-National Botanical Research Institute, INDIA

## Abstract

Brassinosteroids (BRs) hormones are important for plant growth, development and immune responses. They are sensed by the transmembrane receptor kinase *Brassinosteroid-Insensitive 1 (BRI1)* when they bind to its extracellular Leu-rich repeat (LRR) domain. We cloned and characterized the *TaBRI1* from *T*. *aestivum* and raised overexpression transgenics in *Arabidopsis* to decipher its functional role. TaBRI1 protein consists of a putative signal peptide followed by 25 leucine rich repeats (LRR), a transmembrane domain and a C-terminal kinase domain. The analysis determined the interaction of TaBRI1 with five members of the wheat *Somatic Embryogenesis Receptor Kinase* (*TaSERKs*) gene family (TaSERK1, TaSERK2, TaSERK3, TaSERK4 and TaSERK5), at the plasma membrane. Furthermore, overexpression of *TaBRI1* in *Arabidopsis* leads to the early flowering, increased silique size and seed yield. Root growth analysis of *TaBRI1* overexpressing transgenic plants showed hypersensitivity to epi-brassinolide (epi-BL) hormone in a dose-dependent manner. Interestingly, transgenic *Arabidopsis* plants show thermotolerance phenotype at the seedling stages as revealed by chlorophyll content, photosystem II activity and membrane stability. The transcriptome profiling on the basis of microarray analysis indicates up-regulation of several genes related to brassinosteroid signaling pathway, abiotic stress response, defense response and transcription factors. These studies predict the possible role of *TaBRI1* gene in plant growth and development imparting tolerance to thermal stress.

## Introduction

Plant growth regulators brassinosteroids (BRs) are ubiquitously present throughout the plant kingdom and play a pivotal role in plant growth and development with regulatory functions during cell elongation, cell division, vascular differentiation, biotic and abiotic stress response and senescence [1, 2]. BRs are sensed by the *brassinosteroid-insensitive 1* (*BRI1*), a member of the leucine rich repeat receptor kinase (LRR-RK) family, at the cell surface for the coordinated action in response to changing environment [3]. *BRI1* gene was first identified and characterized in *Arabidopsis* as a BR insensitive mutant [4]. In recent years, it was isolated from various plant species, including dicots viz., *Pisum sativum* [5], *Lycopersicon esculentum* [6], *Gossypium hirsutum* [7] and *Glycine max* [8] and monocots such as *Oryza sativa* [9] and *Hordeum vulgare* [10]. *BRI1* is ubiquitously expressed in almost all organs in *Arabidopsis* and rice and is localized to the plasma membrane [9, 11]. The strongest evidence for the indispensable nature of BRs as plant hormones comes from the discovery of *Arabidopsis thaliana* mutants in the mid 1990s resulting in the loss of BR biosynthesis and having characteristic dwarf stature with curled leaves, short stems, delayed senescence and flowering and de-etiolated phenotypes [12–14]. In the past few years, studies on *BRI1* highlights its valuable role in imparting plant architecture and yield enhancement [8]. In *O*. *sativa*, the BRI1 loss of function mutant depicted reduction in height significantly with little effect on fertility [15]. In *H*. *vulgare*, the *BRI1* mutant showed similar BR insensitive mutant phenotype which leads to lodging resistance [10]. All these investigations develops our interest to study the BRI1 function in wheat which is one of the major crop plant.

Brassinosteroid signal transduction involves several upstream and downstream signaling components which have been studied in detail. A small group of RLKs namely Somatic Embryogenesis Receptor Kinases (SERKs) show close homology with the Brassinosteroid-Insensitive1 (BRI1) Associated Receptor Kinase 1 (BAK1) and BAK1-like 1 (BKK1) which act as co-receptors in the BR signal transduction. Besides its BR function, BAK1 plays a role in several pathways independently by enhancing the signal output of varied LRR-RLK partners through binding with different ligands [16, 17]. In the absence of BR, BRI1 appear to exist in plasma membranes as a ligand independent homodimer whose cytoplasmic domain interacts with BRI1 Kinase Inhibitor 1 (BKI1) inhibiting the interaction between the BRI1 and its co-receptor Somatic Embryogenesis Receptor Kinase (SERK) subfamily of LRR-RLKs/ BRI1-Associated Receptor Kinase I (BAK1) [17–19]. The binding of ligand-receptor induces conformational change leading to autophosphorylation of the BRI1 kinase domain which further creates a docking platform for the interaction of BRI1 with its co-receptor SERKs/BAK1 [20–22]. Signal transduction to BR responses is mediated by plasma membrane-associated BR-signaling kinases (BSKs) which inhibit the kinase activity of a major downstream negative regulator BIN2 by promoting the BSU1 (BRI1 suppressor 1) phosphatase [23, 24]. BIN2 inactivation causes the activation of two closely related transcription factors, Brassinazole-Resistant 1(BZR1) and BRI1-Ems Suppressor 1 (BES1) in nuclei, eliciting a wide range of gene expression changes of BR responses [25, 26].

We report here the characterization of *Brassinosteroid insensitive 1*, *TaBRI1* gene isolated from *Triticum aestivum* cDNA library [27]. In this report we analyze its structural organization and create a phylogenetic tree. We explored the protein-protein interactions of TaBRI1 with its coreceptors, TaSERKs. Our results show that *TaBRI1* overexpressing transgenic lines in *Arabidopsis* display faster germination, early flowering, longer siliques and higher seed yield. *TaBRI1* results in enhanced root growth which is inhibited in a concentration dependent manner in presence of BR. To provide additional evidence we checked its response during thermal stresses.

## Materials and Methods

### Plant material and growth conditions

*Arabidopsis thaliana* ecotype Col-0 plants was used as wild-type (WT) as genetic background to raise transgenic plants and for all experiments. Seeds were obtained from Arabidopsis Biological Resource Centre, USA. Plants were grown under 16 h light (~100–125 μmol m^-2^s^-1^ photoperiod) and 8 h dark at 22±1°C, in pots containing soilrite supplemented with nutrient medium [28]. *Arabidopsis* seeds were surface sterilized with 2% sodium hypochlorite and 0.01% Triton X-100, washed with sterile RO water and inoculated in petriplates containing MS medium (pH 5.8) supplemented with 2% sucrose and 0.8% agar which were then placed to cold room (4°C) for stratification for two days followed by transfer to culture room. For expression analysis, 3-week-old seedlings were treated with different hormones (BL, auxin, BAP and ABA) at various intervals, frozen in liquid nitrogen and stored at -70°C until further use.

### Sequence analysis of *TaBRI1*

The full length cDNA sequence was used to search homologous sequences and percentage sequence similarity via BLASTX and BLASTP in NCBI database (http://www.ncbi.nlm.nih.gov). For domain analysis TaBRI1 protein sequence was analyzed by CDD (Conserved domain database) and SMART (Simple Modular Architecture Research Tool). Multiple sequence alignment was carried out by CLUSTALW program and phylogenetic tree was constructed using MEGA6 program by neighbor-joining method.

### Subcellular localization of TaB2 protein coding gene

The complete open reading frame (ORF) of TaB2 protein coding gene was fused to yellow fluorescent protein (YFP) reporter gene in frame under the control of the cauliflower mosaic virus 35S promoter (CaMV 35S) in pSITE–3CA vector. About 2μg of the plasmid construct was used to coat gold particles. Inner epidermal peels of white onion were placed inside–up on MS medium. Onion (*Allium cepa*) peels were bombarded by using PDS–1000/ He system (Bio–Rad, Canada) at 1,100 p.s.i. with DNA coated gold particles and 6cm of target distance using 1.0μm of gold micro–carriers. After bombardment, the petriplates were sealed with parafilm and incubated overnight at 28°C before observation. The onion epidermal cells were observed by using confocal microscope (Leica ^®^ TCS SP5II).

### *TaBRI1* cloning and overexpression in *Arabidopsis*

For generating overexpression construct of *TaBRI1* gene to characterize its function, a 3.4 Kb ORF was amplified with gene specific primer (S1 Table) using cDNA as a template isolated from 13-d-old leaf base of *T*. *aestivum*. The amplified product was then cloned in an entry vector (pENTR^™^/ D-TOPO) and then in destination vector pMDC32 under CaMV 35S promoter following Gateway ^™^ cloning strategy (Directional TOPO Cloning kit and LR clonase Enzyme mix II kit, Invitrogen Inc. USA). The AGL1 strain of *Agrobacterium tumefaciens* harboring pMDC32-*TaBRI1* was used for transformation in *Arabidopsis* through floral dip method [29]. The T1 seeds were selected on MS-agar plates supplemented with 50 μg/μl hygromycin and the resistant plants transferred to pots. All further analysis was conducted using best three transgenic lines (T4 seeds) homozygous for the transgene.

### Yeast two hybrid analysis

From pENTR/D-TOPO vector the ORF of TaBRI1 was cloned into bait vector pDEST-GBKT7 (DNA binding domain, BD) and all five TaSERKs (activation domain, AD) into prey vector pDEST-GADT7 using Gateway^™^ LR Clonase^™^ enzyme mix (Invitrogen). Both the construct were co-transformed into yeast strain AH109 and selected on SD medium lacking Trp and Leu, respectively, as per the manufacturer protocol. Further, inoculum from primary culture containing colonies of SD (-L,T) plates were used in the secondary culture (3 mL) and grown at 30°C, 200 rpm for 3 h till the OD at 600 nm reached 0.5. Each culture was then serially diluted (10^−1^, 10^−2^, 10^−3^ and 10^−4^) and droplets of 10μL of each dilution including the undiluted culture were placed on the selection media (SD/-LT and SD/-HLT supplemented with 1mM 3-aminotriazole) and incubated at 30°C for 3–5 days till the formation of colonies.

### Bimolecular fluorescence complementation (BiFC) assay and intracellular localization

To visualize protein-protein interaction BiFC assay was performed. Cloning of TaBRI1 and TaSERKs was done in Gateway^™^ vectors pSITE-nEYFP and pSITE-cEYFP, complete ORF (without stop codon) were amplified using Phusion High Fidelity Taq Polymerase (Finnzymes) and inserted into the entry vector (pENTR^™^/D-TOPO) following the above BiFC destination vector using Gateway ^™^ LR Clonase enzyme mix (Invitrogen Inc. USA) as per the manufacturer protocol. For visualization of YFP fluorescence these constructs particle bombarded on onion epidermal cells using Biolistic PDS– 1000/He particle delivery system (Bio- Rad, USA) according to the protocol described earlier [30]. 3 μg of DNA was used for each construct to coat 0.2 mg gold particles per shot in a chamber vaccum of 27 in Hg pressure where particles accelerated with 1100 psi from the cylinder containing helium (He) gas pressure and the onion peel plate was loaded on to the target platform (6 cm from the launch assembly). The plates were incubated at 28°C in dark conditions for 12–16 h for stabilization. The onion peels were then observed for YFP expression under the Leica^®^ TCS SP5II confocal microscope.

### Root growth inhibition assays

For root growth analysis in presence of brassinosteroid as well as abiotic stresses, treatment with epi-brassinolide (24 epi-BL) purchased from Sigma (St Louis, MO, USA) was used. Twenty seedlings were grown on half-strength MS medium for 3-days and then transferred on fresh MS plates supplemented with different working concentrations of epi-BL which were added from 10^−2^ M stock solution in 95% ethanol before the media solidified. Petriplates containing seedlings were grown vertically in culture room under 16 h light and 8 h dark at 22±1°C for 4 days. The root length was measured on fifth day of transfer and compared with respect to control (MS medium without hormone). In addition to determine the effect of heat and cold stress, 7-day old seedlings grown on control conditions were given heat stress at 40°C for 2 h and cold stress at 4°C for 24 h. Plates were then kept vertically for five days in culture room and root length, plant height as well as rosette diameter examined after fifth day. All experiments were done in triplicates and the values presented in the data are mean of these experiments. Standard error and student’s t-test was used to display significant difference between WT and transgenic and a *P*-value ≤ 0.05.

### Physiological analysis of overexpression transgenics

#### Estimation of total chlorophyll content

Chlorophyll content was estimated from the non- stressed and stressed 21-d old seedlings (twenty seedlings each) by using 100 mg of tissue for both WT and transgenic lines in each tube containing 2.5 mL of DMSO. The tubes were then incubated overnight in dark for chlorophyll leaching. Absorbance was recorded at 645 and 663 nm in a UV-Vis spectrophotometer (Agilent Cary 60). Chlorophyll content was calculated according to the formula of Arnon [31].

#### Photosynthetic Yield (Fv/Fm)

Photosystem II activity was recorded of WT and transgenic leaves under stressed and non-stressed conditions using a pulse amplitude modulation fluorometer (Junior PAM-210, H. Waltz, Germany) at room temperature. Chlorophyll fluorescence measurements were made from the upper surface of the leaves which were dark-adapted for 20 min before measuring the induction of fluorescence [32]. The intensity of the measuring, modulated red light was ~0.1 μmol.m^-2^ s^-1^to induce the minimum fluorescence (F_0_). Saturating flashes were provided to completely reduce the PSII acceptor site Q_A_- and to measure the maximum fluorescence yield (F_m_). The intensity of the saturating light flash (1s) used for the measurements of Fm was 3000 μmol.m^-2^ s^-1^. Photosynthetic yield was measured using the following formula: Fv/Fm where Fv = Fm-Fo [33]. The ratio F_v_: F_m_ reflects the potential yield of the photochemical reaction of PSII.

#### Membrane Stability Index (MSI)

MSI was determined by recording the electrical conductivity according to the Sairam *et al*. [34] protocol. 21-d old stressed and non-stressed seedling tissue (0.1g) was taken in double distilled water and initial conductivity (C1) measured at 30°C for 30 min. The samples were then autoclaved for 15 min. and again readings were measured termed as C2 by conductivity meter (Eutech instrument, Singapore). MSI was calculated using formula: MSI = {1-(C1/C2)}*100

### Real Time PCR analysis

Total RNA was isolated by RNeasy Plant Mini Kit (Qiagen, Germany) according to the manufacturer protocol followed by on-column DNase-I treatment for removal of genomic DNA contamination. For real time PCR analysis, first strand cDNA was synthesized with 2μg of total RNA using the High Capacity cDNA Archive kit (Applied Biosystems, USA) and 200nM of each primer mixed with SYBR Green PCR Master Mix (Applied Biosystems) for real-time PCR reactions, using the ABI Prism 7000 Sequence Detection System and Software (PE Applied Biosystems) according to the manufacturer’s instruction. Primer Express 2.0 (Applied Biosystems^®^) was used for designing primers from a unique region, specific to the gene and each pair was confirmed by BLAST program in NCBI and TAIR database (S1 Table). The relative mRNA levels in different RNA samples were normalized with respect to internal control gene, *Actin*. The Ct (threshold cycles) values were averaged for two biological replicates and three technical replicates.

### Transcriptome analysis of *TaBRI1* overexpression transgenics

For microarray analysis, approximately 20–30 *Arabidopsis* seedlings of twelve-day old WT overexpression transgenic was harvested, weighed and frozen in liquid nitrogen. The RNA was isolated using RNeasy plant mini kit (Qiagen) and quality and quantity of RNA were checked by Agilent Bioanalyzer (Agilent Technologies, North Carolina, USA). Microarray hybridization was performed using Affymetrix GeneChip 3′-IVT express kit according to manufacturer's instructions (Affymetrix, Santa Clara, CA, USA) with 500 ng RNA. Affymetrix gene chip data files (.CEL files) were generated and analysed by ARRAY ASSIST software where normalization of data and probe summarization for all the genes on the chip was done by Gene Chip Robust Multiarray Analysis (GCRMA) algorithm. This was followed by log transformation of data and finally average log signal intensity values of three biological replicates were computed with overall correlation coefficient values of ≥ 0.95 were used for analyzing differential gene expression. Genes showing up- and down-regulation with a fold change ≥ 2 and a *p*-value cut off ≤0.05 was selected with respect to the wild-type. The heat maps showing the expression profiles of varied genes were generated using signal intensity values via Multi Experiment Viewer (v4.8).

### Statistical analysis

Statistical analysis was done by calculating mean value and standard error (SE) for all the three replicates. Student’s t-test was performed to reveal significant differences between WT and transgenic lines. A *P*-value of ≤0.05 was considered significant.

## Results

### Sequence, structural analysis and sub-cellular localization of TaBRI1

**Fig 1:**
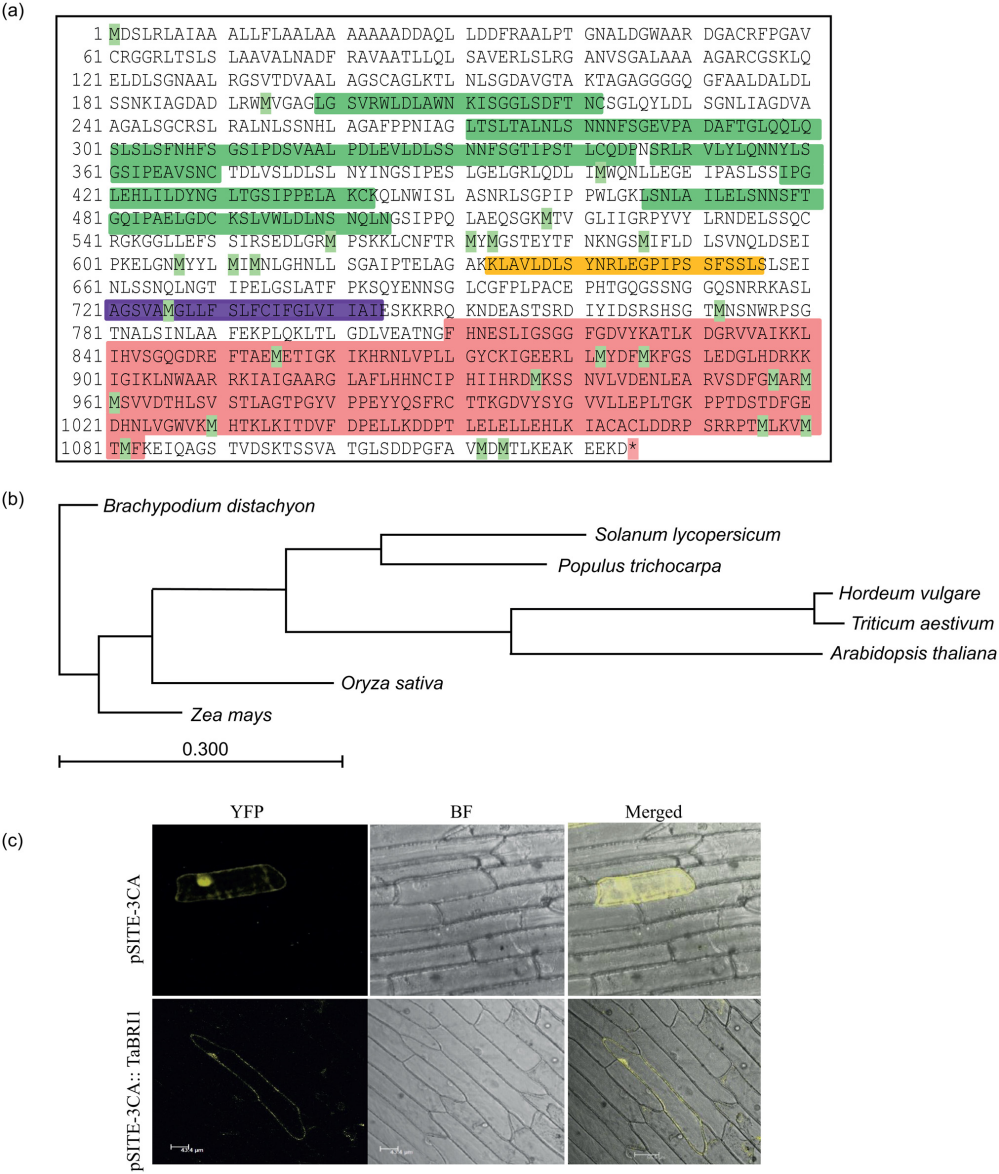
Sequence and structural analysis of *TaBRI1*. (a) Schematic representation of protein sequence of TaBRI1, domains are highlighted viz. LRR domain (green), LRR_1 (yellow), transmembrane (purple) and kinase domain (pink). (b) Phylogenetic tree of *T*. *aestivum*, TaBRI1 homologs from different plant species. The bar represents number of amino acid substitutions per site. (c) Sub-cellular localization of TaBRI1. Images were monitored in YFP filter and merged by confocal microscopy after 24 h of incubation. The chimeric protein was localized to the nucleus as well as in the plasma membrane while in control, YFP is detected throughout the cell. YFP (Yellow fluorescent protein); BF (Bright field).

With the aim to elucidate the molecular and functional role of *TaBRI1*, an EST of 1.5 kb of *TaBRI1* gene was identified from 13-day-old leaf base, auxin induced, cDNA library from wheat, *T*. *aestivum* [27]. Full length of *TaBRI1* (DQ655711.1 Accession no.) 3,834 bp was amplified and the ORF of 3.4 Kb was then cloned in overexpression vector pMDC32 under CaMV 35S promoter. The deduced protein comprises of 1,124 amino acid residues with a predicted molecular weight of 119.81 kDa and an isoelectric point of 5.69. TaBRI1 protein sequence revealed that its polypeptide (Fig 1a) harbours 25 leucine rich repeat (LRR) from position 199 to 514 amino acid (green boxes) among which eight LRR domains are highly conserved that are a characteristic feature of BR receptor, PFAM LRR_1 domain from position 633 to 655 amino acid (yellow box) suggested to be involved in diverse protein-protein interactions, followed by a transmembrane region from position 721 to 743 amino acid (purple box) and a long stretch of kinase _Tyr domain from position 810 to 1083 amino acid (pink box) comprising of serine/threonine kinase and tyrosine protein kinase at C- terminal end that possibly takes part in most of the cellular activities. The multiple sequence alignment of *Triticum aestivum*, TaBRI1 showed high homology to other BRI1 of various plant species (S1 Fig). Based on the BLASTP from NCBI database TaBRI1 showed 96% similarity with HvBRI1 homolog of *Hordeum vulgare* (Accession No. BAD01654.1) highlighting the close relatedness, 83% identity with homolog of *Oryza sativa*, OsBRI1 (Accession No. NP001044077.1), 79% similarity with the homolog of *Zea mays*, ZmBRI1 (Accession No.XP008656807.1) and 49% identity with the putative homolog of *Brachypodium distachyon*, BdBRI1 (Accession No. XP003577946.1). It also showed homology with dicot plant species where TaBRI1 shares 57% similarity with the putative homolog of *Solanum lycopersicum*, SlBRI1 (Accession No. XP004237477.1), 55% similarity with homologs of other two dicots *Populus trichocarpa*, PtBRI1 (Accession No. XP002307140.2) and *Arabidopsis thaliana*, AtBRI1 (Accession No. NP195650.1).

The phylogenetic tree constructed revealed that *T*. *aestivum*, TaBRI1 shares maximum closeness with *H*. *vulgare*, HvBRI1 (Fig 1b). As observed the tree does not show a clear demarcation between monocots and dicots clustering. Among the dicot plant species, *S*. *lycopersicum*, SlBRI1 and *P*. *trichocarpa*, PtBRI1 grouped in separate clade and clustered together showing close relatedness with each other. However, *A*. *thaliana*, AtBRI1 arise from the same branch node as observed in TaBRI1 and HvBRI1 which suggests they might be closely related with respect to their evolution. Indeed, from the sequence alignment and phylogenetic tree of *T*. *aestivum*, TaBRI1 depicts high degree of conservation implicating this gene might play an important role as a BR receptor in the brassinosteroid transduction pathway.

Transient expression assays in onion epidermal cells by confocal microscopy revealed that 35S:TaBRI1-YFP fusion protein was mainly expressed in the nucleus and plasma membrane as compared to the YFP alone which was found to be distributed throughout the cell (Fig 1c).

### *In vivo* identification of TaBRI1 interaction with TaSERKs

**Fig 2:**
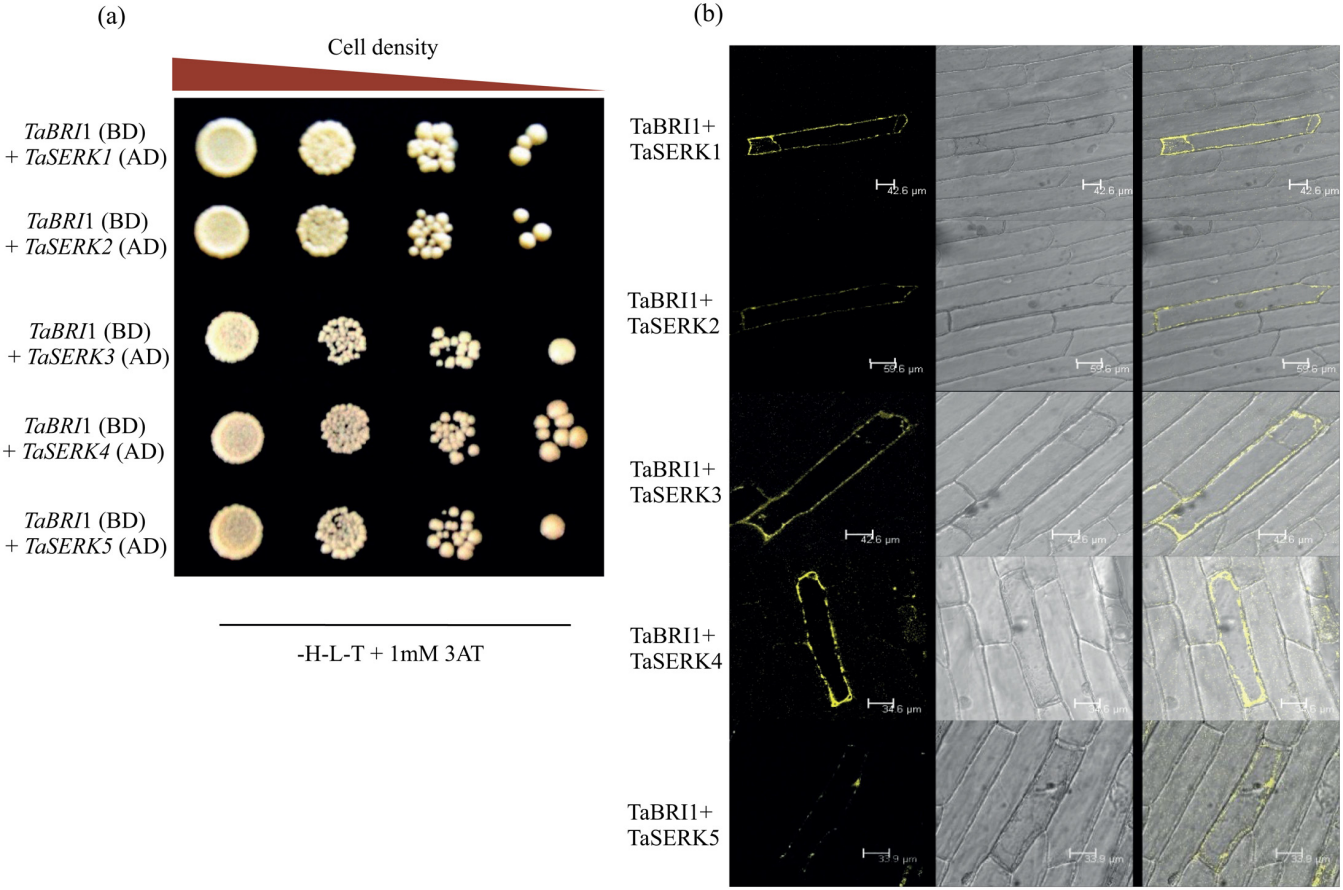
Identification of TaBRI1 interaction with TaSERKs. (a) *in vivo* interaction tests in yeasts. TaBRI1 protein fused to DNA- binding domain (TaBRI1-pGBKT7) used as a bait to examine its interaction with other protein named as TaSERKs fused to the activation domain (TaSERKs-pGADT7) as a prey. The co-transformed yeast with these constructs on drop-out medium lacking histidine, leucine and tryptophan in the presence of 1mM 3-aminotriazole. (b) BiFC visualization of TaBRI1 interaction with TaSERKs (TaSERK1, TaSERK2, TaSERK3, TaSERK4 and TaSERK5) transiently expressed in onion epidermal cells. YFP fluorescence and merged images of epidermal cells shown in this image confirmed positive interaction as expressed in plasma membrane.

Several reports showed hetero-oligomerization of BRI1 receptor and BAK1 (SERK3), SERK1 and SERK4 required in BR signaling mechanism in *Arabidopsis* [35, 36]. We examined the protein-protein interactions between *T*. *aestivum*, TaBRI1 and TaSERKs (TaSERK1, TaSERK2, TaSERK3, TaSERK4 and TaSERK5) using yeast two-hybrid system. The interactions of TaBRI1 with the TaSERK family members were specific as revealed by the presence of viable colonies (Fig 2a) on the selection medium (SD -H,L,T). Also, visualization of fluorescence in the plasma membrane performed by BiFC assay (Fig 2b) showed the positive interaction of TaBRI1 with the five TaSERKs. These results, therefore conclude that TaBRI1 heterodimerizes with the TaSERKs viz. TaSERK1, TaSERK2, TaSERK3, TaSERK4 and TaSERK5.

### Overexpression of *TaBRI1* causes early flowering in *Arabidopsis* transgenics

**Fig 3:**
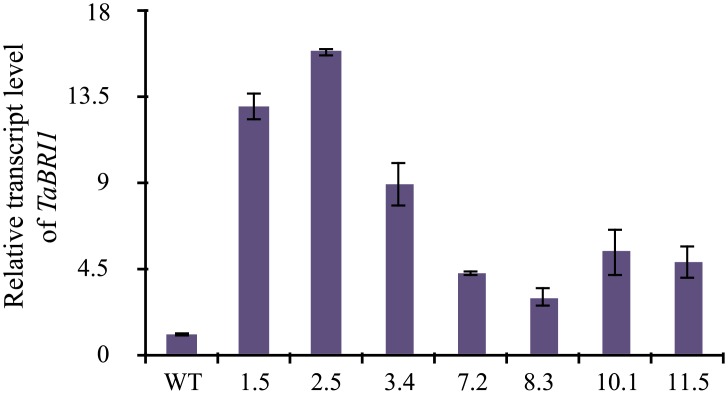
Overexpression of *TaBRI1* in transgenic *Arabidopsis* plants. Real-time PCR analysis of wild-type (WT) and *TaBRI1* transgenic *Arabidopsis* plants, showing the expression in different homozygous transgenic lines (line 1.5, 2.5, 3.4, 7.2, 8.3, 10.1 and 11.5) with little expression in wild-type. *Actin* gene was used as an internal control. Data are mean ± SE from two biological and three technical replicates.

**Fig 4:**
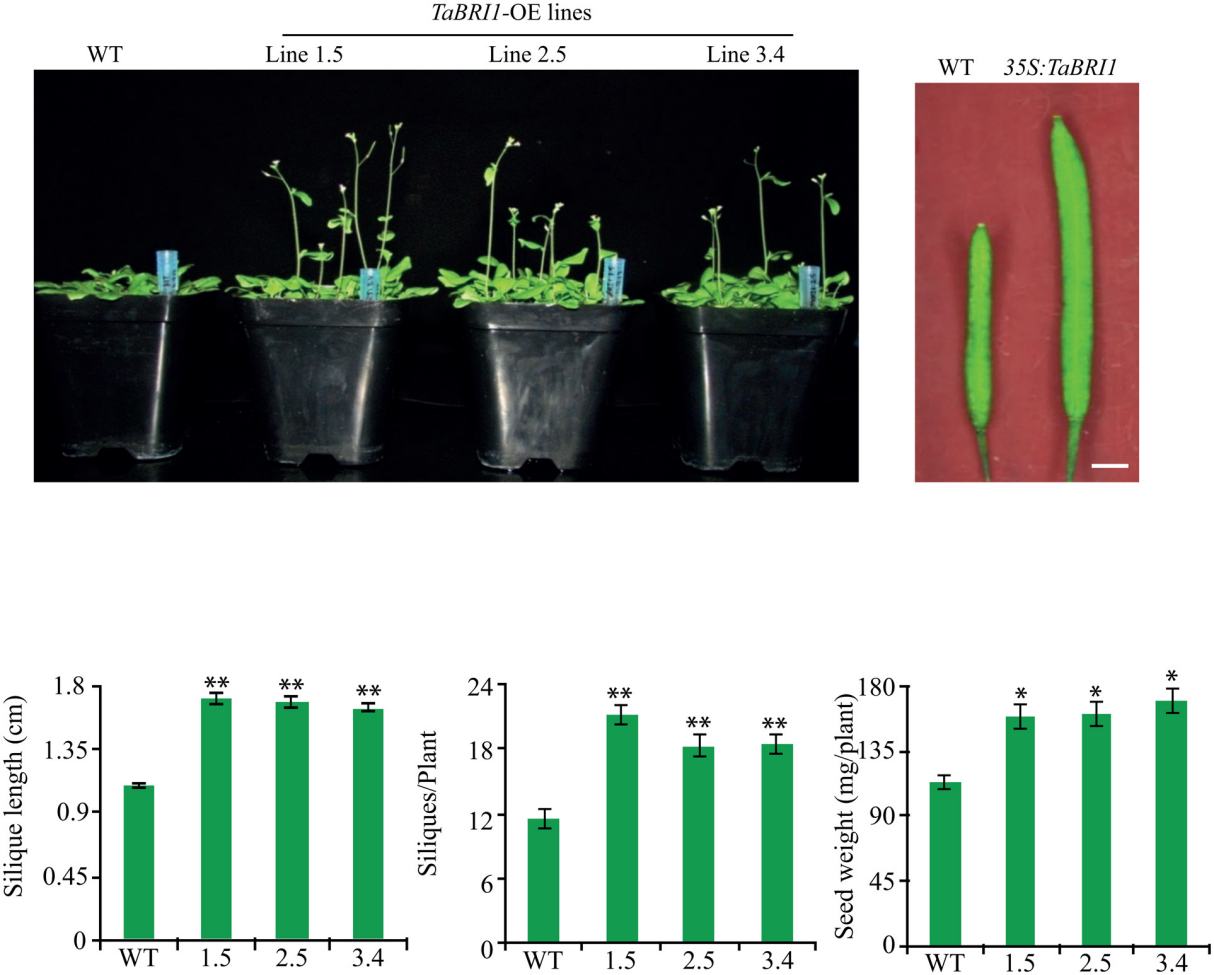
Phenotype of *TaBRI1* overexpression transgenic lines in *Arabidopsis thaliana*. (a) *TaBRI1* transgenic lines showed early flowering as compared to the Col-0 WT grown under 16 h light and 8 h dark culture condition at the same time. (b) Two month old overexpression lines had larger siliques than wild-type. (c) Graphical representation of silique length (d) total number of siliques per plant. (e) Seed weight per individual plant (N = 10). Data represents mean ±SE and data points marked with asterisk (**P ≤ 0.01 and *P ≤ 0.05) indicate statistically significant differences between WT and overexpression transgenic lines.

Expression of transgenic lines were examined in seven independent lines (1.5, 2.5, 3.4, 7.2, 8.3, 10.1 and 11.5) by real-time PCR analysis (Fig 3) where the transgenic lines showed varying transcript levels relative to the wild-type (WT). Three transgenic lines (1.5, 2.5 and 3.4) which exhibit an increased expression levels were selected for further analysis. To explore the role of *TaBRI1*, *Arabidopsis* transgenic *TaBRI1*-OE and Col-0 WT were grown under long day culture conditions of 16 h light and 8 h dark. Transgenic *TaBRI1* plants displayed early flowering as compared to the WT (Fig 4a). Flowering time was recorded as the number of days from the transfer of plants to the culture room to the day of the appearance of floral buds. *TaBRI1* overexpression transgenic plants started flowering 10–12 days earlier than the WT as monitored and recorded in Table 1. Also, the constitutive expression of *TaBRI1* in *Arabidopsis* resulted in larger silique size (Fig 4b and 4c) and increased number of siliques per plant than that of WT (Fig 4d) which leads to the enhancement of total seed yield per plant in *TaBRI1* overexpression transgenic plants (Fig 4e). The increased seed production in transgenics was mainly due to a greater number of seeds per plant since no significant difference was observed in seed size as compared to the WT. In addition, enhanced plant height was also observed at 35 days after germination in transgenic *TaBRI1* overexpression plants than the WT (Table 1). However, less number of rosette leaves was monitored in *TaBRI1*-OE plants at the time of bolting as compared to the WT, while, rosette diameter in transgenic lines was similar to the WT which inferred that the flowering time was affected by the transgene activity. Similarly, leaf length and leaf width of *TaBRI1*-OE was comparable with that of WT and no significant difference could be deduced.

**Table 1:** Morphological comparison between *TaBRI1* overexpression (OE) and WT *Arabidopsis* plants.

Morphological features	TaBRI1-OE			
	WT	Line1.5	Line2.5	Line3.4
Average flowering time ^a^ (days)	27.8±1.64	18.8±0.83	19.8±0.83	20.8±1.30
Plant height ^b^ (cm)	20.8±1.64	27.8±1.92	28±1.58	27.6±1.51
Rosette diameter ^c^ (cm)	5±0.23	5.22±0.25	5.3±0.15	5±0.12
Rosette leaf number ^c^	12.6±1.34	9.2±0.83	9.0±1.00	9.4±1.13
Leaf length ^d^ (cm)	1.98±0.08	2.34±0.43	2.26±0.26	2.12±0.39
Leaf width ^d^ (cm)	0.96±0.11	0.7±0.07	0.78±0.17	0.76±0.08
Seed length (mm)	0.43±0.01	0.5±0.03	0.48±0.04	0.49±0.04
Seed width (mm)	0.26±0.05	0.34±0.04	0.33±0.05	0.32±0.04

^a^ number of days recorded when plants formed floral buds

^b^ maximum height attained by plants after 35 days

^c^ at the time of bolting

^d^ of the largest leaf at the time of bolting.

### *TaBRI1* overexpression leads to hypersensitivity to brassinosteroid mediated root growth

**Fig 5:**
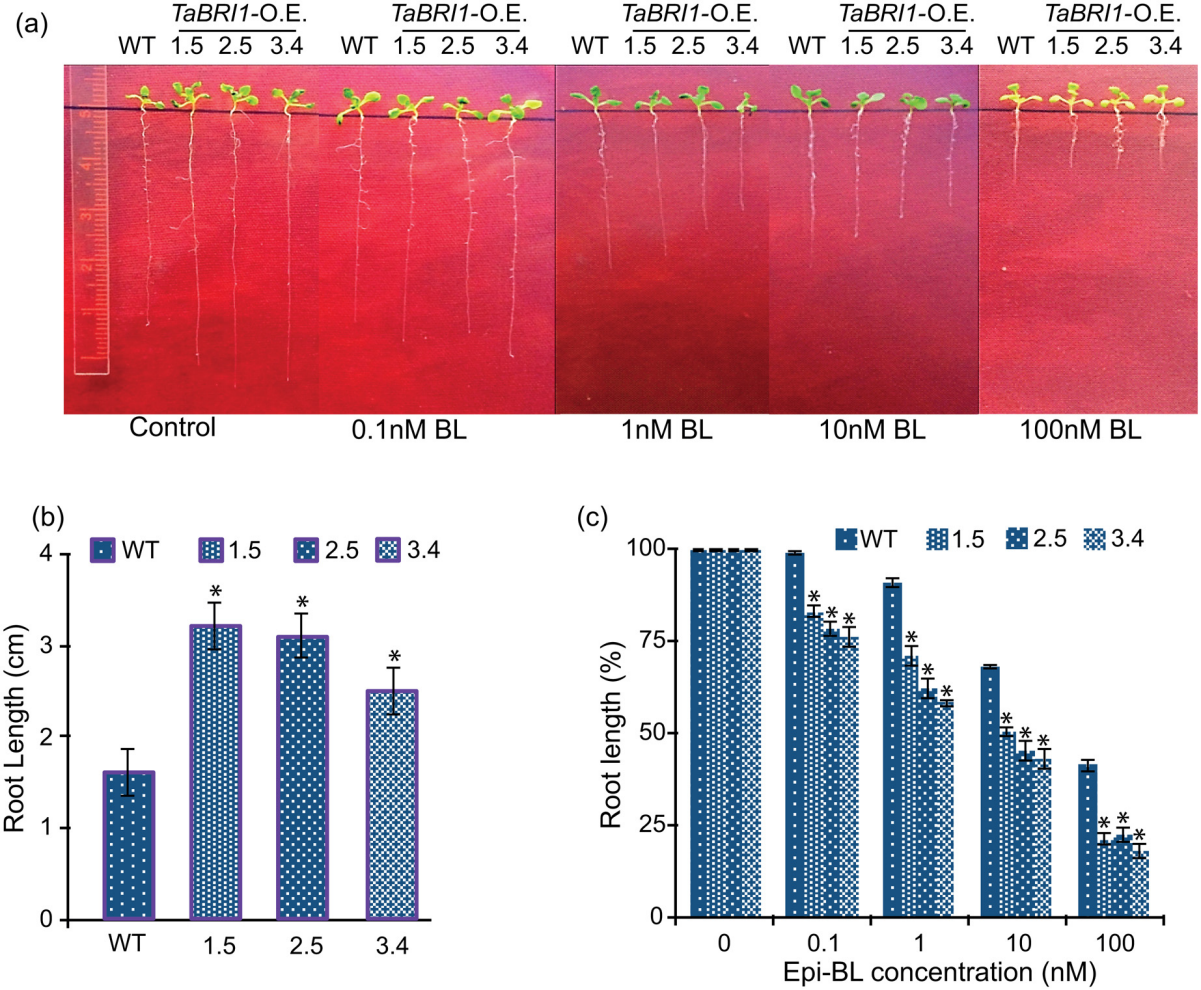
*TaBRI1* overexpression (OE) transgenic lines enhances root growth in *Arabidopsis* plant. (a) Sensitivity of *TaBRI1*-OE transgenics to brassinosteroid (epi-BL) in root growth inhibition assay. 12-d-old seedlings of WT and *TaBRI1*-OE were grown on half-strength MS medium in the presence or absence of the indicated concentration of epi-BL (nM) after germination. (b) Histograms represent the root length of WT and *TaBRI1*-OE (1.5, 2.5 and 3.4) of 10-d old seedlings under control conditions. (c) Root length normalized as a percentage of the untreated mock control for WT and *TaBRI1*-OE transgenic lines on epi-BL (nM) supplementation. Values are mean ± SD for ten seedlings each. Asterisk and double asteriks (**P ≤ 0.01 and *P ≤ 0.05) indicate statistically significant differences between WT and overexpression transgenic lines.

At lower concentrations BRs play an indispensable role in the growth and development of roots [37]. In order to determine how exogenously applied epi-brassinolide (BL) affects root growth, we investigated the response of WT and *TaBRI1*-OE *Arabidopsis* seedlings on different concentrations of BL. *TaBRI1* overexpression *Arabidopsis* seedlings were more sensitive to the brassinosteroid hormone and result in root length inhibition in a concentration dependent manner (Fig 5a). The overexpression *TaBRI1* lines showed enhanced primary root growth under control conditions (without BR treatment) as compared to the WT (Fig 5b) however, root length gradually decreased as the concentration of BL increases from 0.1 nM to 100 nM from 100% to 20% at 100 nM epi-BL whereas in WT the decrease in primary root length was from 100% to 40% (Fig 5c). These results imply hypersensitivity of overexpression *TaBRI1* transgenics in presence of exogenously supplied BL.

### *TaBRI1* imparts heat and cold stress tolerance to transgenic *Arabidopsis*

**Fig 6:**
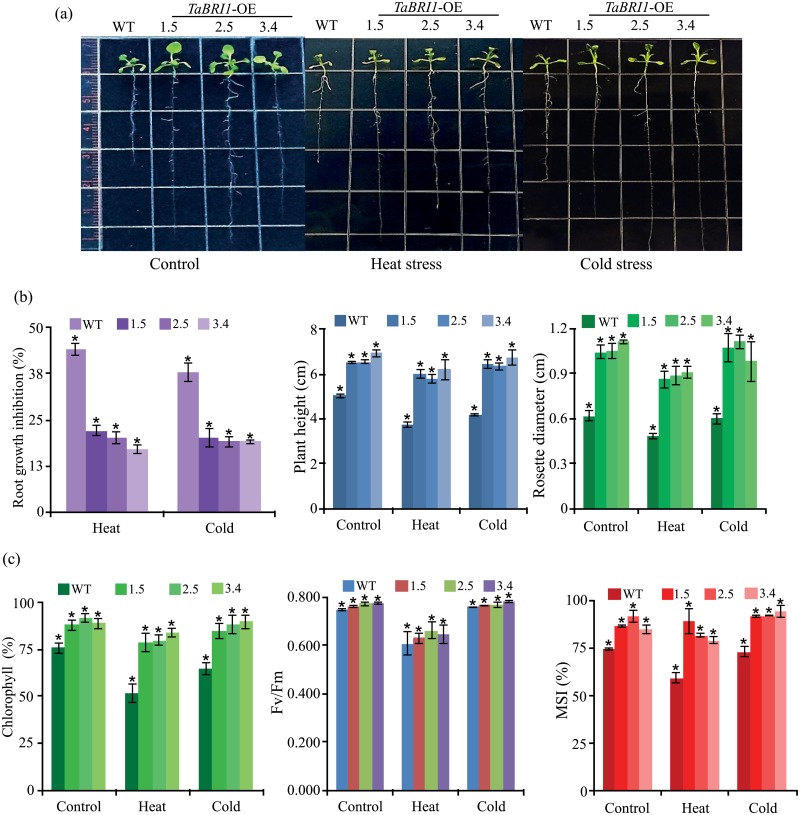
Effect of heat and cold stress on WT and *TaBRI1* overexpression transgenic lines in *Arabidopsis*. (a) Phenotype of root growth of 12-d-old WT and transgenics grown on half-strength MS medium after heat stress for 2 h at 40°C and cold stress at 4°C for 24 h given to 7-d-old seedlings. Comparison of (b) root growth inhibition, plant height and rosette diameter under control, heat and cold stress conditions. (c) Physiological parameters viz. chlorophyll content, Fv/Fm and membrane stability index (MSI) under heat and cold stress between WT and transgenic lines. Values are mean ± SE from three independent experiments. Asterisk and double asteriks (**P ≤ 0.01 and *P ≤ 0.05) indicate statistically significant differences between WT and overexpression transgenic lines.

To analyze the response of transgenics to temperature extremes, the *TaBRI1* transgenics and WT were grown on half-strength MS medium for 7 days under control conditions. For heat stress, the plates were incubated at 40°C for 2 h and at 4°C for 24 h for cold stress and transferred to the culture room for 5 days. Data presented in Fig 6a and 6b shows, the transgenic *TaBRI1*-OE lines exhibited elongated roots as compared to the WT, and the inhibition in root growth in different transgenic lines was lower under heat and cold stress as compared to WT. Also, an increase in plant height and rosette diameter of *TaBRI1*-OE transgenic lines was observed (Fig 6b) resulting in an overall tolerant phenotype (remained green, healthier and decreased senescence) under heat and cold stress conditions. In addition to the morphological analysis, we also monitored the stress tolerance levels quantitatively by analyzing physiological parameters like chlorophyll content, photosynthetic efficiency and membrane stability. Under non-stressed conditions, the level of chlorophyll content in overexpression lines of *TaBRI1* showed higher levels as compared to the WT (Fig 6c) however, transgenics under heat and cold stress conditions had higher chlorophyll levels as compared to WT. Additionally, we observed the Fv/Fm ratio to check the photosynthetic efficiency (PSII), where the activity increases in overexpression lines under non-stressed and stressed conditions (Fig 6c) and also significant differences were observed on membrane stability index (MSI) (Fig 6c) where the percentage of MSI under heat stress and cold stress conditions increased to 70–80% as compared to the WT, respectively. The above results implicate enhanced expression of *TaBRI1* leads to better temperature tolerance than the WT plants via maintaining membrane integrity, hence this gene can be exploited for heat and cold stress tolerance.

### Whole genome transcriptome profiling of *TaBRI1* overexpression transgenics

**Fig 7:**
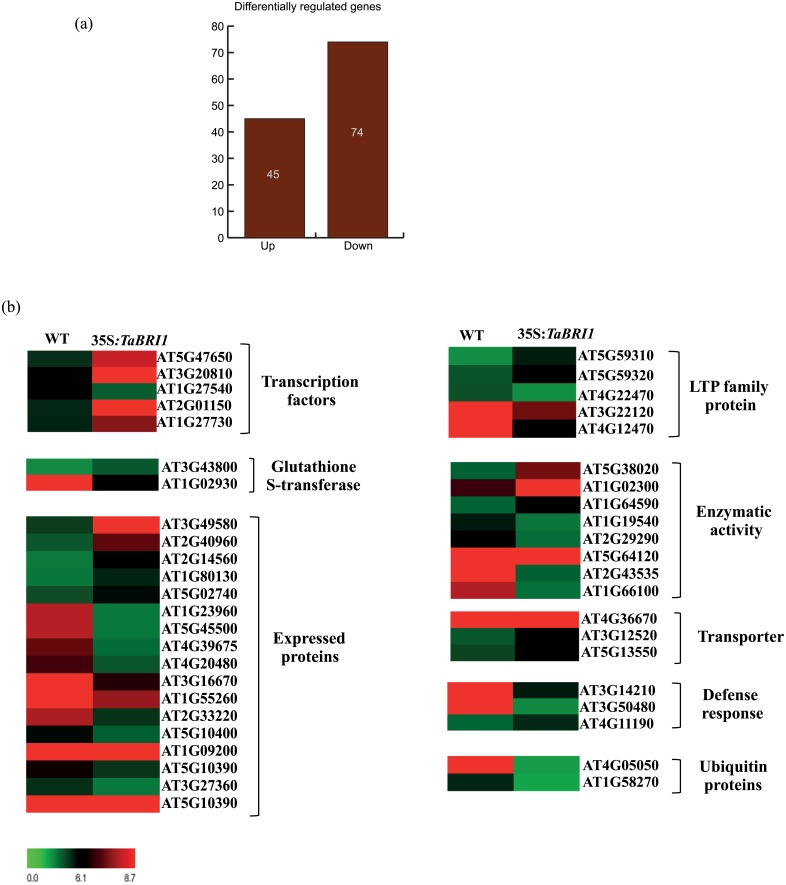
Differential gene expression in wild-type and overexpression transgenic lines. Heat maps showing the differential expression of selected genes involved in diverse biological processes and molecular functions in transgenic lines as compared to the WT under control conditions.

In an attempt to understand the fundamental mechanism causing the above mentioned morphological changes and study the hypersensitive response towards brassinolide hormone, whole genome microarray analysis of WT and transgenic seedlings (line 1.5) was performed. Data analysis revealed that a total of 119 genes (≥ 2 fold change and p value ≤ 0.05) were differentially expressed in transgenic line constitutively expressing *TaBRI1* with respect to the WT, out of which 45 were up-regulated and 74 down-regulated (S2 and S3 Tables). This included the genes encoding proteins which acts as transcription factor families like F-Box, Zinc finger (C2H2 and C3HC4 type) and jumonji (jmjc), stress responsive proteins like glutathione S-transferase (GST tau and phi type), putative expressed proteins, LTP protein family, genes involved in enzymatic activity like lipooxygenase (LOX2), carboxymethyl transferase, protease, reductase, peroxidase, dehydrogenase, cytochrome P450 (CP450), putative thionine and trypsin inhibitor, transporter proteins like sulphate and mannitol transporter, senescence and defense response related genes (CC-NBS-LRR and LRR) and ubiquitin proteins (Fig 7). Amongst these many play an important biological role in growth and development of plants, abiotic stress response, disease resistance and proteasomal degradation.

**Fig 8:**
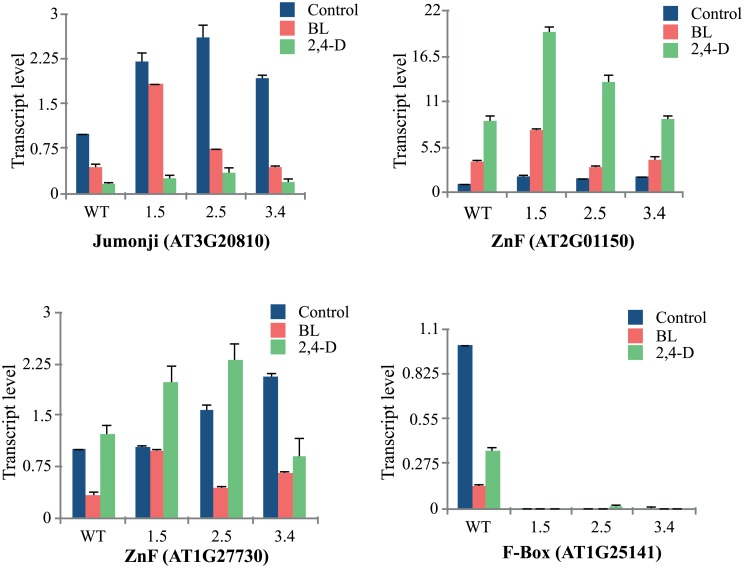
Real-time PCR analysis showing differential expression of transcription factors in presence of epi-brassinolide (BL, 1μM) and auxin (2,4-D, 10 μM) treated for 1 h.

**Fig 9:**
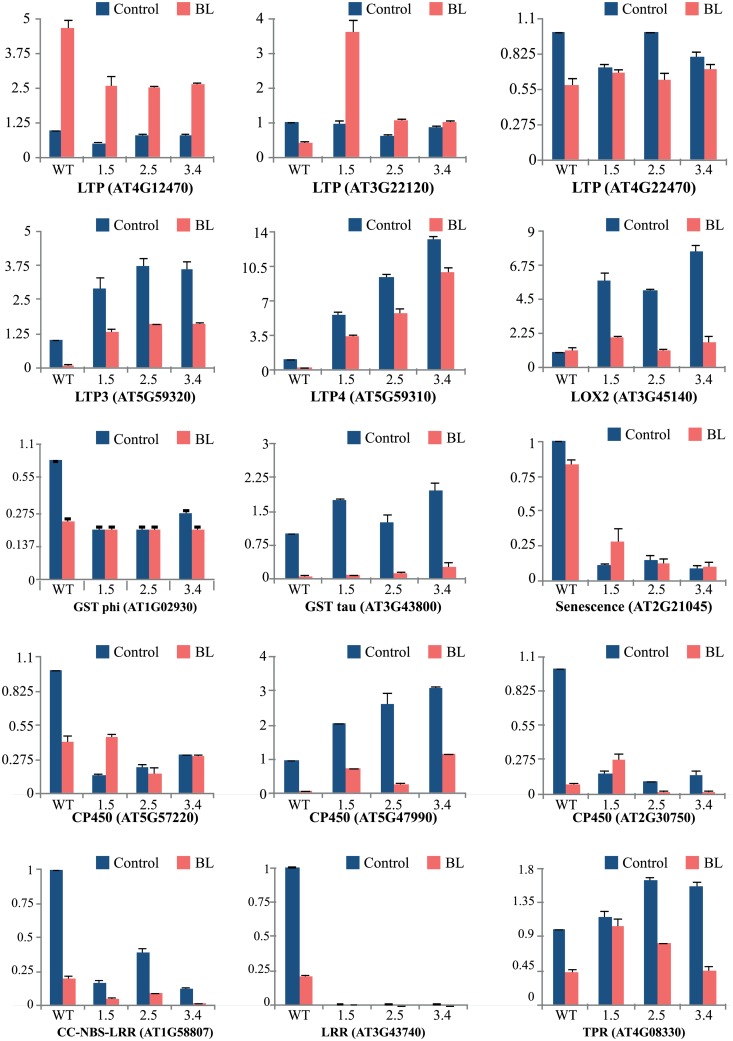
Real-time PCR analysis showing differential expression of genes selected from microarray analysis in transgenic lines and WT *Arabidopsis* seedlings in presence of epi-brassinolide (BL, 1μM) treated for 1 h.

To validate the results obtained from the microarray analysis we checked the transcript levels of above mentioned transcription factors in presence of either both brassinosteroid (epi-BL) or auxin (2,4-D) and also investigated some of the other interesting up- and down-regulated genes influenced by brassinosteroid (epi-BL) through real-time PCR analysis. The real-time PCR data validated the observation by microarray analysis thus reconfirming the differential regulation of genes in response to BR and 2,4-D. Among the transcription factors, only zinc finger was found to be auxin mediated showing a fold change of >20 in transgenics, whereas no significant difference was observed in presence of BR. However, transcript levels of other transcription factors viz., jumonji and F-Box do not show a significant difference in presence of hormones (Fig 8). Further, genes which are related to the other biological functions such as plant development, stress response, defense related and enzymatic activity also showed varied transcript levels in control as well as in BL-treated seedlings (Fig 9). The transcript levels for *LTP3* and *LTP4* were higher in *TaBRI1*-OE seedlings while other putative *LTPs* show down-regulation in transgenics with no significant difference as compared to the WT. Additionally, the results obtained for transcripts of *LOX2*, *GST* (tau), *CP450* (Accession no. AT5G47990) and *TPR* shows upregulation in transgenics when compared with WT, were consistent with the microarray data. In contrast, the transcript levels of *GST* (phi), senescence related gene, other *CP450* genes, *CC-NBS-LRR* and *LRR* down-regulated in *TaBRI1*-OE seedlings whereas in presence of BR overall decrease in the transcript levels of selected genes was observed as compared to the control conditions, respectively.

## Discussion

We describe here the isolation and characterization of *TaBRI1* gene from *T*. *aestivum*, most closely related to the *H*. *vulgare*, *HvBRI1*. Sequence analysis revealed that the TaBRI1 protein contains 25 LRR among which eight LRRs are highly conserved and kinase domains at its carboxyl terminal which is a characteristic feature of BR receptors required for BRI1 function in BR signal transduction pathway [4]. TaBRI1 interacts with five TaSERKs isolated from *T*. aestivum which acts as a homolog of BAK1 coreceptor in the BR signaling pathway. Ectopic expression of *TaBRI1* leads to early flowering in *Arabidopsis* and enhances root growth. *TaBRI1* gene expression was differentially regulated by hormones and is hypersensitive in presence of BR in a dose-dependent manner. Transgenic *Arabidopsis* plants overexpressing *TaBRI1* showed considerable tolerance to adverse temperature stresses. These results imply that *TaBRI1* is a novel gene which plays crucial role in plant growth and development and participate in abiotic stress tolerance.

### TaBRI1 shares conserved domains with other plant species

The predicted TaBRI1 protein shares conserved domain structure with other BRI1 polypeptides which include leucine rich repeats (LRR) followed by PFAM LRR_1 domain and a serine (Ser)/ threonine (Thr) kinase domain at the carboxyl terminal which is responsible for BR reception in the BR signal transduction pathway [4]. TaBRI1 shows strong sequence similarity with *H*. *vulgare*, HvBRI1 (96%) and OsBRI1 of *O*. *sativa*, (83%) and phylogenetic relationship displayed that TaBRI1 group together with HvBRI1 in the same clade. *Brassinosteroid-Insensitive 1* (*BRI1*) was originally identified from *Arabidopsis* mutant analysis which was then cloned and demonstrated for the presence of leucine rich repeat receptor like kinase [4] located specifically in the plasma membrane [11, 38]. Similarly, in the present study also TaBRI1 is also found to be localized to the plasma membrane.

### Protein-protein interaction of TaBRI1 with TaSERKs

Many studies have shown the *in vivo* interaction between BRI1, a ligand dependent receptor of BR signaling with members of the Somatic Embryogenesis Receptor like Kinase (SERK) subfamily [39]. SERK3 homolog of BRI1-Associated Receptor Kinase 1 (BAK1) acts as a coreceptor show interaction with BRI1 both *in vitro* and *in vivo* in *Arabidopsis* [18, 40, 41]. SERK1, also heterodimerizes with BRI1 to enhance BR signaling [42]. SERK4, also known as BAK1-LIKE (BKK1), shows interaction with BRI1 *in vivo* in a BR-dependent manner [43]. Our study shows the *in vivo* interaction of TaBRI1 with all five identified TaSERKs (TaSERK1, TaSERK2, TaSERK3, TaSERK4 and TaSERK5) at the plasma membrane where TaBRI1 oligomerizes with respective TaSERKs independent of the BR ligand. Since interaction of both the receptors is found to be necessary in the initiation of BR signaling pathway, it would be interesting to explore further the co-localization of TaBRI1 and TaSERKs in presence of BRs.

### Ectopic overexpression of *TaBRI1* causes early flowering and root growth development

BR’s have a pleiotropic effect on the vegetative and reproductive development of plants which is mediated by BZR1 regulation by reducing transcript levels of a potent floral repressor [44]. The present results shed light on reproductive development and root growth. BR insensitive mutants and *bri1* mutants were reported to be involved in late flowering [4, 12]. Moreover, BRI1 was found to be an important player participating as a flowering time enhancer by repressing FLOWERING LOCUS C (FLC) expression, a MADS-box transcription factor [45]. Also, other components of BR signal transduction, BES1 interacting proteins, ELF6 (early flowering 6), its homolog REF6 (relative of early flowering 6) and JMJ30 which belongs to the Jumonji N/C (jmjN/C) domain containing proteins were found to regulate time of flowering [46, 47]. These studies imply a strong connection between BR signal transduction and pathways controlling floral initiation [48]. The present study provides evidence that ectopic expression of *TaBRI1* in *Arabidopsis* results in early flowering of transgenics and enhances the seed yield via increase in number of siliques.

Roots are one of the major sources of BRs which play an important role in plant growth and development. Several BR-deficient mutants display impaired root growth and display a short root phenotype than the wild-type. However, effect of BR on root growth depends on its concentration being stimulative at low levels and inhibitory at high levels [49, 50]. In the present study, we demonstrate that overexpression of *TaBRI1* enhances primary root growth as compared to the WT while, inhibition of root length occurs in overexpressing lines as the concentration of BL increases indicating hypersensitivity of primary root in a dose dependent manner. In support of our results, transgenic plants ectopically overexpressing the BAK1-associating receptor-like kinase 1(BARK1) have enhanced primary root growth and show hypersensitivity to BR in a concentration dependent manner [51].

### *TaBRI1* imparts abiotic stress tolerance

Besides the well characterized function of *BRI1* during growth and development their biological role in response to abiotic stress tolerance remain to be explored. The expression of *OsBRI1* found to be upregulated under salinity stress conditions [52]. In contrast to our finding, mutant *bri1-9* was found to be more resistant to cold stress than the *BRI1* overexpressing *Arabidopsis* plants [53]. To gain insights on the role of *TaBRI1* gene during environmental stress we investigated the root length phenotype of *TaBRI1* overexpressing transgenics in presence of heat and cold stress. Root growth assays provide both qualitative and quantitative data of plants to various stresses [54]. In our study we observed decreased root growth inhibition, enhanced plant height and high chlorophyll content, photosynthetic activity and better membrane stability in transgenic lines under heat and cold stress as compared to WT. These results suggested that *TaBRI1* might be involved in thermal stress tolerance and can be considered as a suitable gene to improve crop plants under extreme environmental stress conditions.

### Transcriptome analysis of *TaBRI1* transgenics identify differentially expressed genes

The whole- genome expression profiling of *TaBRI1* overexpression revealed differential expression of several transcription factors, amongst which jumonji (jmjc), zinc finger (C3HC4 type) and zinc finger (C2H2 type) were up-regulated while F-box transcription factor was down-regulated. One of the reports showed that putative zinc finger transcription factor (AT4G39070) was repressed by BR hormone, thus negatively regulating the BR signaling pathway. Our real time PCR analysis also showed that the putative zinc finger transcription factor (AT1G27730) showed down-regulation of transcript level in presence of BR while in presence of auxin it was upregulated, which means this zinc-finger response is auxin-mediated. In contrast, one putative zinc finger transcription factor (AT2G01150) was upregulated both in the presence of BR and auxin and a jumonji N/C domain- containing transcription factor was involved in reproductive development by directly interacting with BES1. RELATIVE OF EARLY FLOWERING (REF6), a jumonji transcriptional regulator, represses FLOWERING LOCUS C (FLC) thereby leading to early flowering by BR-mediated signal transduction [47, 46]. In the present study, the expression of jumonji transcription factor in transgenics decreases in presence of BR and auxin as compared to the control condition (without hormone treatment). Further, F-box transcription factor which acts as a receptor and signaling component in plant hormone signaling pathway showed downregulation in transgenics and almost negligible expression in presence of BR and auxins.

We also analyzed several development, stress and defense related genes like Lipid Transfer Protein (LTP) family protein, Lipoxygenase (LOX2), Glutathione S- transferase (GST, tau and phi class), senescence associated family protein, Cytochrome P450, CC-NBS-LRR, LRR and TPR. Earlier reports showed that *LTP* gene family plays an important role in plant growth and development, defense against pathogens, as well as adaptation of plants towards various environmental stresses [55]. In this study, five putative *LTP*s were differentially expressed, where *LTP3* and *LTP4* were up-regulated in transgenics, other putative LTPs were down-regulated. These were selected and validated in presence of BL by real-time PCR analysis. Influence of hormones was observed in only one of the putative *LTP* (AT4G12470) whereas no significant change was apparent in other *LTP*s which suggests that their regulation is not BR mediated. *LOX2*, encodes a chloroplast lipoxygenase plays a key role in wound induced synthesis of the plant growth regulator jasmonic acid, JA [56]. Analysis of *LOX2* done by Müssig *et al*. [57] revealed that transcript levels enhanced on application of BR in WT and BR- deficient mutants (*cbb1/dwf1*) which explains BR and JA interaction might be conditional during plant development. However, in our studies *LOX2* gene showed higher fold change in transgenics and is down-regulated on application of BL. The Cytochrome P450, implicated to be an essential player of BR biosynthesis, however, accession number AT5G57220 encodes CYP81F2 which is involved in aphid resistance [58] downregulated in *TaBRI1* transgenics and At5G47990 encoding CYP705A5 upregulated in transgenics was known to be involved in regulating gravitropism [59]. Moreover, the *TaBRI1* overexpression may be detrimental in R-gene mediated resistance since the expression of *CC-NBS-LRR* class gene [60] was downregulated in transgenics. Tetratricopeptide repeat (TPR) domains domains which are involved in mediating protein-protein interactions are present in many stress related genes and in steroid receptor complexes [61] found to be upregulated in *TaBRI1* transgenic. TPR domains are present at the C-terminus of BSK1 and BSK2 (BR signaling kinases) sequence which might act as a substrate of BRI1 or its coreceptor BAK1 [62].

Overall, our results demonstrate that constitutive expression of *TaBRI1* leads to early flowering, modulates root development and confer tolerance under adverse temperature stress. In addition, TaBRI1 shows *in vivo* interaction with all five TaSERKs isolated from wheat at the plasma membrane. In future, it will be of interest to investigate protein-protein interaction between TaBRI1 and TaSERKs in presence of BR and to understand the intricacies of BR-dependent and BR-independent pathways in higher plants.

## Supporting Information

S1 Fig Multiple sequence alignment of amino acid sequences of TaBRI1 (*Triticum aestivum*) with other organisms. The different domains are shown by colored boxes which are marked on the top of each domain.
(PPTX)

S1 Table List of primers used in the experiment. (DOCX)

S2 Table List of upregulated genes obtained from microarray analysis. (DOCX)

S3 Table List of downregulated genes obtained from microarray analysis. (DOCX)

## References

[1] Clouse SD, Sasse JM. Brassinosteroids: essential regulators of plant growth and development. Ann Rev Plant Physiol Plant Mol Biol. 1998; 49: 427–451. 10.1146/annurev.arplant.49.1.427 15012241

[2] Mandava BN. Plant growth promoting brassinosteroids. Ann Rev Plant Physiol Plant Mol Biol. 1988; 39: 23–52. 10.1146/annurev.pp.39.060188.000323

[3] Belkhadir Y, Chory J. Brassinosteroid signaling: a paradigm for steroid hormone signaling from the cell surface. Science. 2006; 314: 1410–1411. 10.1126/science.1134040 17138891

[4] Li J, Chory J. A putative leucine-rich repeat receptor kinase involved in brassinosteroid signal transduction. Cell. 1997; 90: 929–938. 10.1016/S0092-8674(00)80357-8 9298904

[5] Nomura T, Bishop GJ, Kaneta T, Reid JB, Chory J, Yokota T. The *LKA* gene is a *BRASSINOSTEROID INSENSITIVE 1* homolog of pea. Plant J. 2003; 36: 291–300. 10.1046/j.1365-313X.2003.01863.x 14617087

[6] Montoya T, Nomura T, Farrar K, Kaneta T, Yokota T, Bishop GJ. Cloning the tomato *curl3* gene highlights the putative dual role of the leucine-rich repeat receptor kinase tbri1/sr160 in plant steroid hormone and peptide hormone signaling. Plant Cell. 2002; 14: 3163–3176. 10.1105/tpc.006379 12468734 PMC151209

[7] Sun Y, Fokar M, Asami T, Yoshida S, Allen RD. Characterization of the brassinosteroid insensitive 1 genes of cotton. Plant Mol Biol. 2004; 54: 221–232. 10.1023/B:PLAN.0000028788.96381.47 15159624

[8] Wang M, Sun S, Wu C, Han T, Wang Q. Isolation and characterization of the brassinosteroid receptor gene (*GmBRI1*) from *Glycine max*. Int J Mol Sci. 2014; 15: 3871–3888. 10.3390/ijms15033871 24599079 PMC3975373

[9] Yamamuro C, Ihara Y, Wu X, Noguchi T, Fujioka S, Takatsuto S, et al Loss of function of a rice brassinosteroid insensitive1 homolog prevents internode elongation and bending of the lamina joint. Plant Cell. 2000; 12: 1591–1605. 10.1105/tpc.12.9.1591 11006334 PMC149072

[10] Chono M, Honda I, Zeniya H, Yoneyama K, Saisho D, Takeda K, et al A semidwarf phenotype of barley uzu results from a nucleotide substitution in the gene encoding a putative brassinosteroid receptor. Plant Physiol. 2003; 133: 1209–1219. 10.1104/pp.103.026195 14551335 PMC281616

[11] Friedrichsen DM, Joazeiro C, Li J, Hunter T, Chory J. Brassinosteroid-insensitive-1 is a ubiquitously expressed leucine-rich repeat receptor serine/ threonine kinase. Plant Physiol. 2008; 123: 1247–1256. 10.1104/pp.123.4.1247 PMC59084 10938344

[12] Chory J, Nagpal P, Peto CA. Phenotypic and genetic analysis of det2, a new mutant that affects light-regulated seeding development in *Arabidopsis*. Plant Cell. 1991; 3: 445–459. 10.1105/tpc.3.5.445 12324600 PMC160013

[13] Li J, Nagpal P, Vitart V, McMorris TC, Chory J. A role for brassinosteroids in light-dependent development of *Arabidopsis*. Science. 1996; 272: 398–401. 10.1126/science.272.5260.398 8602526

[14] Szekeres M, Nemeth K, Koncz-Kalman Z, Mathur J, Kauschmann A, Altmann T, et al Brassinosteroids rescue the deficiency of CYP90, a cytochrome P450, controlling cell elongation and de-etiolation in *Arabidopsis*. Cell. 1996; 85: 171–182. 10.1016/S0092-8674(00)81094-6 8612270

[15] Morinaka Y, Sakamoto T, Inukai Y, Agetsuma M, Kitano H, Ashikari M, et al Morphological alteration caused by brassinosteroid insensitivity increases the biomass and grain production of rice. Plant Physiol. 2006; 141: 924–931. 10.1104/pp.106.077081 16714407 PMC1489896

[16] Chinchilla D, Shan L, He P, de Vries S, Kemmerling B. One for all: The receptor-associated kinase BAK1. Trends Plant Sci. 2009; 14: 535–541. 10.1016/j.tplants.2009.08.002 19748302 PMC4391746

[17] Clouse SD. Brassinosteroid signal transduction: from receptor kinase activation to transcriptional networks regulating plant development. Plant Cell. 2011; 23: 1219–1230. 10.1105/tpc.111.084475 21505068 PMC3101532

[18] Russinova E, Borst JW, Kwaaitaal M, Cano-Delgado A, Yin Y, Chory J, et al Heterodimerization and endocytosis of Arabidopsis brassinosteroid receptors BRI1 and AtSERK3 (BAK1). Plant Cell. 2004; 16: 3216–3229. 10.1105/tpc.104.025387 15548744 PMC535869

[19] Wang X, Chory J. Brassinosteroids regulate dissociation of BKI1, a negative regulator of BRI1 signaling, from the plasma membrane. Science. 2006; 313: 1118–1122. 10.1126/science.1127593 16857903

[20] Hothorn M, Belkhadir Y, Dreux M, Dabi T, Noel JP, Wilson IA, et al Structural basis of steroid hormone perception by the receptor kinase BRI1. Nature. 2011; 474: 467–471. 10.1038/nature10153 21666665 PMC3280218

[21] Santiago J, Henzler C, Hothorn M. Molecular mechanism for plant steroid receptor activation by somatic embryogenesis co-receptor kinases. Science. 2013; 341: 889–892. 10.1126/science.1242468 23929946

[22] She J, Han Z, Kim TW, Wang J, Cheng W, Chang J, et al Structural insight into brassinosteroid perception by BRI1. Nature. 2011; 474: 472–776. 10.1038/nature10178 21666666 PMC4019668

[23] Kim TW, Guan S, Sun Y, Deng Z, Tang W, Shang JX, et al Brassinosteroid signal transduction from cell-surface receptor kinases to nuclear transcription factors. Nature Cell Biol. 2009; 11: 1254–1260. 10.1038/ncb1970 19734888 PMC2910619

[24] Li J, Nam KH. Regulation of brassinosteroid signaling by a GSK3/SHAGGY-like kinase. Science. 2002; 295: 1299–1301. 10.1126/science.1065769 11847343

[25] Sun Y, Fan XY, Cao DM, Tang W, He K, Zhu JY, et al Integration of brassinosteroid signal transduction with the transcription network for plant growth regulation in *Arabidopsis*. Dev Cell. 2010; 19: 765–777. 10.1016/j.devcel.2010.10.010 21074725 PMC3018842

[26] Yin Y, Wang ZY, Mora-Garcia S, Li J, Yoshida S, Asami T, et al BES1 accumulates in the nucleus in response to brassinosteroids to regulate gene expression and promote stem elongation. Cell. 2002; 109: 181–191. 10.1016/S0092-8674(02)00721-3 12007405

[27] Singla B, Tyagi AK, Khurana JP, Khurana P. Analysis of expression profile of selected genes expressed during auxin-induced somatic embryogenesis in leaf base system of wheat (*Triticum aestivum*) and their possible interactions. Plant Mol Biol. 2007; 65: 677–692. 10.1007/s11103-007-9234-z 17849219

[28] Somerville CR, Ogren WL. Isolation of photorespiration mutants in *Arabidopsis thaliana* In Edelman M., Hollick RB, Chua NH, eds, methods in chloroplast molecular biology. Elsevier Biomedical Press, New York 1982; 129–139.

[29] Clough SJ, Bent AF. Floral dip: a simplified method for *Agrobacterium*-mediated transformation of *Arabidopsis thaliana*. The Plant J. 1998; 16: 735–743. 10.1046/j.1365-313x.1998.00343.x 10069079

[30] Lee LY, Fang MJ, kuang LY, Gelvin SB. Vectors for multi-color bimolecular fluorescence complementation to investigate protein-protein interactions in living plant cells. Plant Methods. 2008; 4: 24 10.1186/1746-4811-4-24 18922163 PMC2572157

[31] Arnon DI. Copper enzymes in isolated chloroplasts. Polyphenoloxidase in *Beta vulgaris*. Plant Physiol. 1949; 24: 1–15. 10.1104/pp.24.1.1 16654194 PMC437905

[32] Checker VG, Khurana P. Molecular and functional characterization of mulberry EST encoding *remorin* (*MiREM*) involved in abiotic stress. Plant Cell Rep. 2013; 32: 1729–1741. 10.1007/s00299-013-1483-5 23942844

[33] Krause GH, Weis E. Chlorophyll fluorescence and photosynthesis: The basics. Ann Rev Plant Physiol Plant Mol Biol. 1991; 42: 313–349. 10.1146/annurev.pp.42.060191.001525

[34] Sairam RK, Rao KV, Srivastava GC. Differential response of wheat genotypes to long term salinity stress in relation to oxidative stress, antioxidant activity and osmolyte concentration. Plant Sci. 2002; 163: 1037–1046. 10.1016/S0168-9452(02)00278-9

[35] Bücherl CA, Van Esse GW, Kruis A, Luchtenberg J, Westphal AH, Aker J, et al Visualization of BRI1 and BAK1(SERK3) membrane receptor heterooligomers during brassinosteroid signaling. Plant Physiol. 2013; 162: 1911–1925. 10.1104/pp.113.220152 23796795 PMC3729770

[36] Wang ZY, Bai MY, Oh E, Zhu JY. Brassinosteroid signaling network and regulation of photomorphogenesis. Ann Rev Genet. 2012; 46: 701–724. 10.1146/annurev-genet-102209-163450 23020777

[37] Kim TW, Lee SM, Joo SH, Yun HS, Lee Y, Kaufman PB, et al Elongation and gravitropic responses of *Arabidopsis* roots are regulated by brassinolide and IAA. Plant Cell Environ. 2007; 30: 679–689. 10.1111/j.1365-3040.2007.01659.x 17470144

[38] Wang ZY, Seto H, Fujioka S, Yoshida S, Chory J. BRI1 is a critical component of a plasma-membrane receptor for plant steroids. Nature. 2001; 410: 380–383. 10.1038/35066597 11268216

[39] Hecht V, Vielle-Calzada JP, Hartog MV, Schmidt ED, Boutilier K, Grossniklaus U, et al The *Arabidopsis* SOMATIC EMBRYOGENESIS RECEPTOR KINASE 1 gene is expressed in developing ovules and embryos and enhances embryogenic competence in culture. Plant Physiol. 2001; 127: 803–816. 10.1104/pp.010324 11706164 PMC129253

[40] Albrecht C, Russinova E, Kemmerling B, Kwaaitaal M, de Vries SC. *Arabidopsis* somatic embryogenesis receptor kinase proteins serve brassinosteroid-dependent and -independent signaling pathways. Plant Physiol. 2008; 148: 611–619. 10.1104/pp.108.123216 18667726 PMC2528080

[41] Li J, Wen J, Lease KA, Doke JT, Tax FE, Walker JC. BAK1, an *Arabidopsis* LRR receptor-like protein kinase, interacts with BRI1 and modulates brassinosteroid signaling. Cell. 2002; 110: 213–222. 10.1016/S0092-8674(02)00812-7 12150929

[42] Karlova R, Boeren S, Russinova E, Aker J, Vervoort J, de Vries S. The *Arabidopsis* Somatic Embryogenesis Receptor-Like Kinase1 protein complex includes Brassinosteroid-Insensitive1. Plant Cell. 2006; 18: 625*–*638. 10.1105/tpc.105.039412 PMC1383638 16473966

[43] He K, Gou X, Yuan T, Lin H, Asami T, Yoshida S, et al BAK1 and BKK1 regulate brassinosteroid-dependent growth and brassinosteroid-independent cell-death pathways. Curr Biol. 2007; 17: 1109–1115. 10.1016/j.cub.2007.05.036 17600708

[44] Zhu JY, Juthamas SS, Wang ZY. Brassinosteroid signaling. Development. 2013; 140: 1615–1620. 10.1242/dev.060590 23533170 PMC3621480

[45] Domagalska MA, Schomburg FM, Amasino RM, Vierstra RD, Nagy F, Davis SJ. Attenuation of brassinosteroid signaling enhances FLC expression and delays flowering. Development. 2007; 134: 2841–2850. 10.1242/dev.02866 17611230

[46] Lu SX, Knowles SM, Webb CJ, Celaya RB, Cha C, Si JP, et al The jumonji C domain–containing protein JMJ30 regulates period length in the *Arabidopsis* circadian clock. Plant Physiol. 2011; 155: 906–915. 10.1104/pp.110.167015 21139085 PMC3032475

[47] Yu X, Li L, Li L, Guo M, Chory J, Yin Y. Modulation of brassinosteroid regulated gene expression by Jumonji domain containing proteins ELF6 and REF6 in *Arabidopsis*. Proc Natl Acad Sci USA. 2008; 105: 7618–7623. 10.1073/pnas.0802254105 18467490 PMC2396691

[48] Clouse SD. The molecular intersection of brassinosteroid-regulated growth and flowering in *Arabidopsis*. Proc Natl Acad Sci USA. 2008; 105: 7345–7346. 10.1073/pnas.0803552105 18495930 PMC2396720

[49] Mouchel CF, Briggs GC, Hardtke CS. Natural genetic variation in *Arabidopsis* identifies BREVIS RADIX, a novel regulator of cell proliferation and elongation in the root. Genes Dev. 2004; 18: 700–714. 10.1101/gad.1187704 15031265 PMC387244

[50] Müssig C, Shin GH, Altmann T. Brassinosteroids promote root growth in *Arabidopsis*. Plant Physiol. 2003; 133: 1261–1271. 10.1104/pp.103.028662 14526105 PMC281621

[51] Kim MH, Kim Y, Kim JW, Lee HS, Lee WS, Kim SK, et al Identification of *Arabidopsis* BAK1-associating receptor-like kinase 1 (BARK1) and characterization of its gene expression and brassinosteroid-regulated root phenotypes. Plant Cell Physiol. 2013; 54: 1620–1634. 10.1093/pcp/pct106 23921992

[52] Sharma I, Ching E, Saini S, Bhardwaj R, Pati PK. Exogenous application of brassinosteroid offers tolerance to salinity by altering stress responses in rice variety Pusa Basmati-1. Plant Physiol Biochem. 2013; 69: 17–26. 10.1016/j.plaphy.2013.04.013 23707881

[53] Kim SY, Kim BH, Lim CJ, Lim CO, Nam KH. Constitutive activation of stress-inducible genes in a brassinosteroid-insensitive 1 (bri1) mutant results in higher tolerance to cold. Physiol Plant. 2010; 138: 191–204. 10.1111/j.1399-3054.2009.01304.x 20053182

[54] Liu N, Zhong NQ, Wang GL, Li LJ, Liu XL, He YK, et al Cloning and functional characterization of *PpDBF1* gene encoding a DRE-binding transcription factor from *Physcomitrella patens*. Planta. 2006; 226: 827–838. 10.1007/s00425-007-0529-8 17541631

[55] Kader JC. Lipid-transfer proteins in plants. Ann Rev Plant Physiol Plant Mol Biol. 1996; 47: 627–654. 10.1146/annurev.arplant.47.1.627 15012303

[56] Bell E, Creelman RA, Mullet JE. A chloroplast lipoxygenase is required for wound-induced jasmonic acid accumulation in *Arabidopsis*. Proc Natl Acad Sci USA. 1995; 92: 8675–8679. 10.1073/pnas.92.19.8675 7567995 PMC41029

[57] Müssig C, Lisso J, Garcia JC, Altmann T. Molecular analysis of brassinosteroid action. Plant Biol. 2005; 8: 291–296. 10.1055/s-2005-873043 16807820

[58] Pfalz M, Vogel H, Kroymann J. The gene controlling the indole glucosinolate modifier1 quantitative triat locus alters indole glucosinolate structures and aphid resistance in *Arabidopsis*. Plant Cell. 2009; 21: 985–999. 10.1105/tpc.108.063115 19293369 PMC2671713

[59] Withers JC, Shipp MJ, Rupasinghe SG, Sukumar P, Schuler MA, Muday GK, et al Gravity persistent signal 1 (gps1) reveals novel cytochrome p450s involved in gravitropism1. Am J Bot. 2013; 100: 183–193. 10.3732/ajb.1200436 23284057

[60] Meyers BC, Kozik A, Griego A, Kuang H, Michelmore RW. Genome-wide analysis of NBS-LRR-encoding genes in *Arabidopsis*. Plant Cell. 2003; 15: 809–834. 10.1105/tpc.009308 12671079 PMC152331

[61] Smith DF. Tetratricopeptide repeat cochaperones in steroid receptor complexes. Cell Stress Chaperones. 2004; 9: 109–121. 15497498 10.1379/CSC-31.1 PMC1065291

[62] Tang W, Kim TW, Oses-Prieto JA, Sun Y, Deng Z, Zhu S, et al BSKs mediate signal transduction from the receptor kinase BRI1 in *Arabidopsis*. Science. 2008; 321: 557–560. 10.1126/science.1156973 18653891 PMC2730546

