# Supplementary material for: Wheat Brassinosteroid-Insensitive1 (TaBRI1) Interacts with Members of TaSERK Gene Family and Cause Early Flowering and Seed Yield Enhancement in Arabidopsis
Source: PLoS One. 2016 Jun 20;11(6):e0153273. doi: 10.1371/journal.pone.0153273 (PMC4913921; doi:10.1371/journal.pone.0153273)
Supplement: S1 Fig — The different domains are shown by colored boxes which are marked on the top of each domain. (PPTX) [file pone.0153273.s001.pptx]

## Slide 1
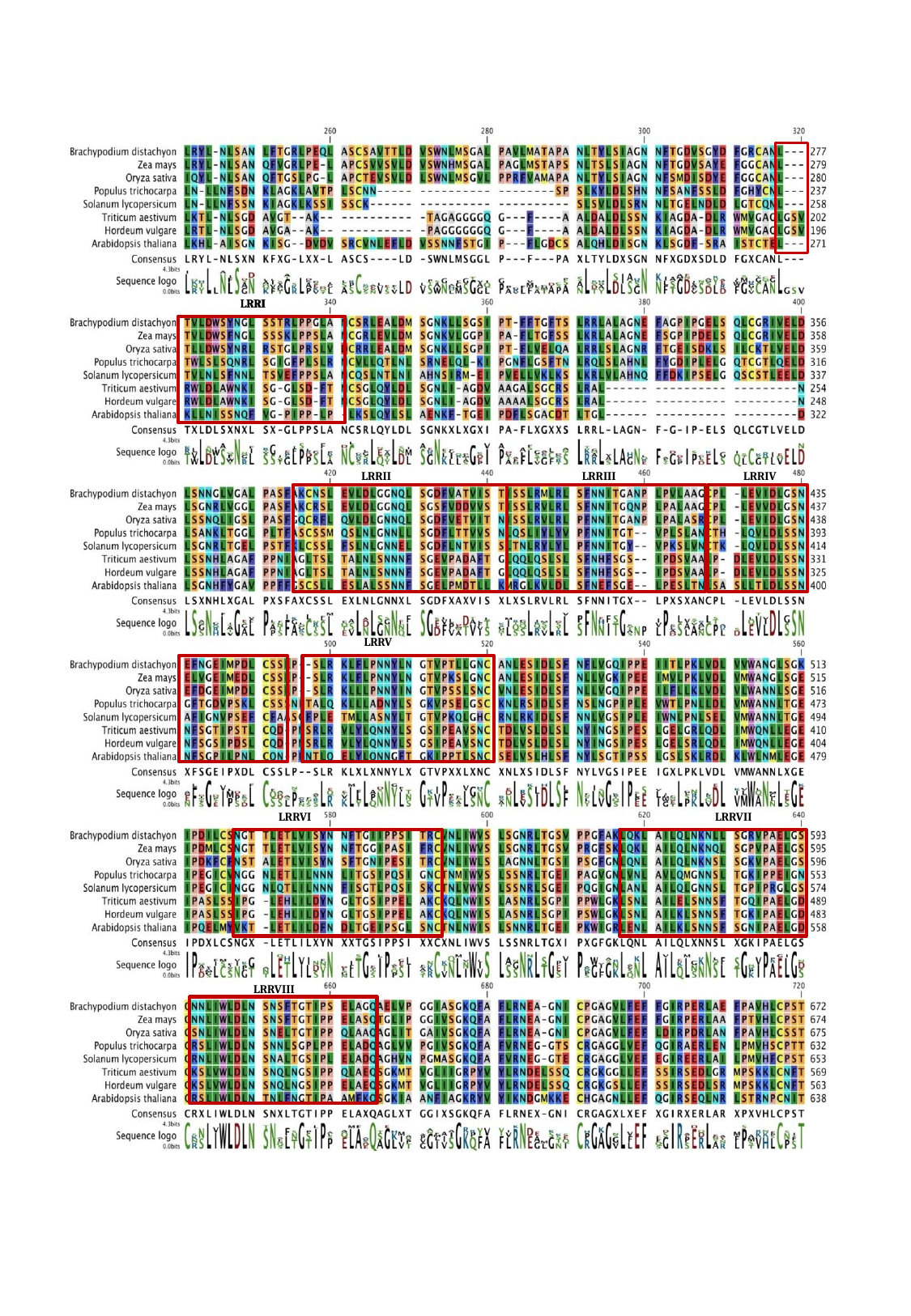

LRRI
LRRII
LRRIII
LRRIV
LRRV
LRRVI
LRRVII
LRRVIII
LRRI

## Slide 2
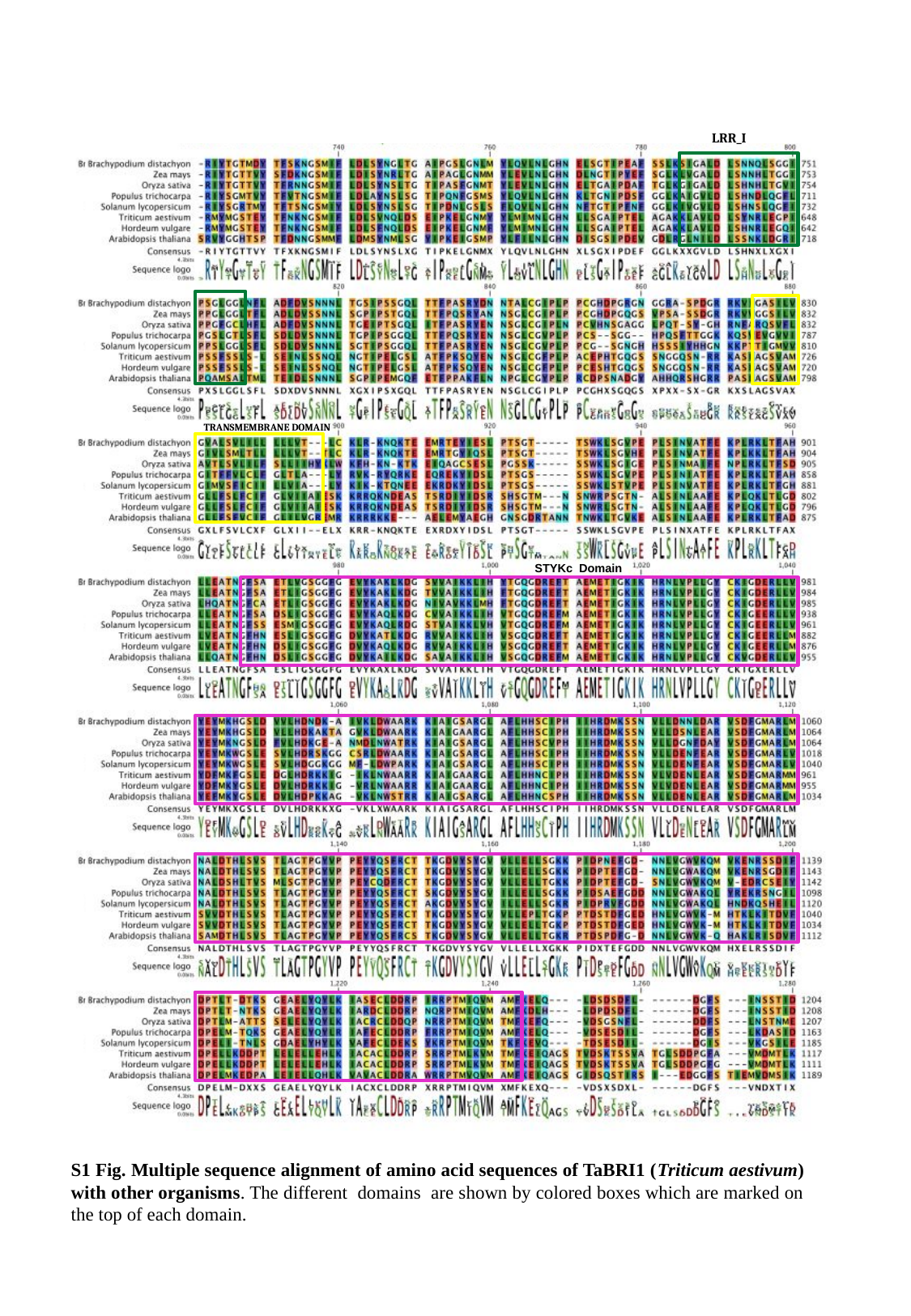

LRR_I
TRANSMEMBRANE DOMAIN
STYKc Domain
TRANSMEMBRANE DOMAIN
STYKc Domain
S1 Fig. Multiple sequence alignment of amino acid sequences of TaBRI1 (Triticum aestivum) with other organisms. The different domains are shown by colored boxes which are marked on the top of each domain.
