# Supplementary material for: Wheat Brassinosteroid-Insensitive1 (TaBRI1) Interacts with Members of TaSERK Gene Family and Cause Early Flowering and Seed Yield Enhancement in Arabidopsis
Source: PLoS One. 2016 Jun 20;11(6):e0153273. doi: 10.1371/journal.pone.0153273 (PMC4913921; doi:10.1371/journal.pone.0153273)
Supplement: S1 Table — (DOCX) [file pone.0153273.s002.docx]

**S1 Table. List of primers used in the experiment**

| **Gene** | **Primer ID** | **Sequence (5’-3’)** |
| --- | --- | --- |
| *TaBRI1* | TOPO-Fwd | CACCATGGATTCCCTGCGGCTGG |
|  | TOPO-Rev | TTCTCCTCCTTGGCTTCCTTGAGGGTCA |
|  | Fwd (Real Time) | CGGAAGATTGCGATAGGATCA |
|  | Rev (Real Time) | TTACTGGATTTCATGTCTCTGTGGAT |
| *Ta-Actin* | Fwd | TTGCACCAAGCAGCATGAA |
|  | Rev | AACCACCGATCCAGACACTGTA |
| *At-Actin* | Fwd | ATCAGCCGTTTTGAATCTCCGG |
|  | Rev | GCCTTTGGGTTAAGAGGAGCCTC |
| ZnF (C3HC4)  [AT1G27730] | Fwd | CAGCTCAACCTAAATCGCCTCTT |
|  | Rev | TAGACAGACACACGATGCAGTCA |
| ZnF (C2H2)  [AT2G01150] | Fwd | GGCGGAGGAGATGATCATTC |
|  | Rev | CGTGTGATTTCCCACTTCCAGTA |
| F-box [AT1G25141] | Fwd | ATGCGTCTGCGTCACCAA |
|  | Rev | AATCCACCTTGTTTGACCAGTACA |
| Jumonji [AT3G20810] | Fwd | CTTCCTCCGGAGACCACAAC |
|  | Rev | TTGCGATTCGGCGTCAA |
| LTP [AT4G12470] | Fwd | CATCGTTAGAGCAGATAACCATTCC |
|  | Rev | CTGGTTGAAATGTTGGTGGAGTT |
| LTP [AT3G22120] | Fwd | CTTCTTCACAAGCAAACATTTCC |
|  | Rev | AGGGTCACTACAATCACAAGCATAAG |
| LTP [AT4G22470] | Fwd | CCATTTCCCTCATCATCATCCT |
|  | Rev | GGTCGGTGTAGGCGAGGCTTA |
| LTP3 [AT5G59320] | Fwd | CCGCCAACAAGCTTGCA |
|  | Rev | GCTAGACTTGGGTTGAGACCAGAA |
| LTP4 [AT5G59310] | Fwd | TGAACGGTATGGCTCAAACCA |
|  | Rev | CTTTTGCAGCGGACTGTAAGC |

| **Gene** | **Primer ID** | **Sequence (5’-3’)** |
| --- | --- | --- |
| LOX2 [AT3G45140] | Fwd | GCATCCTCATTTCCGCTACAC |
|  | Rev | CCACCTCCGTTGACAAGACTTT |
| *GST* [AT1G02930] | Fwd | CAAAGATGGTGAACACAAGAAAGAG |
|  | Rev | CTCCATCTTCAAAGGCTGGAACT |
| *GST* [AT3G43800] | Fwd | TGGGAGACTATTGGTGGTTTTAGTG |
|  | Rev | GGGTCGGGTTAAGCATTTACG |
| Senescence[AT2G21045] | Fwd | CATGGAGGAAACAAAACCAAAGAC |
|  | Rev | CATGACTCTTGGCAAATTCTTCAT |
| Cytochrome P450  [AT5G57220] | Fwd | GCTGAAAAATACGGCGACATC |
|  | Rev | GGCAATGAAGAGATCACTACGACTT |
| Cytochrome P450  [AT5G47990] | Fwd | CCTCGCGCGTGAGATCTTAC |
|  | Rev | TACGGAACCGTTGAAAGAGGAT |
| Cytochrome P450  [AT2G30750] | Fwd | CGCTTCTTTAACTTCCCCATAGTC |
|  | Rev | CTTGGGCCTTGAAGATCTCGTA |
| CC-NBS-LRR  [AT1G58807] | Fwd | CGATTGTTGGAAACGTCTAAGTCAT |
|  | Rev | CGCATGGCGACACTCTCAT |
| TPR [AT4G08330] | Fwd | TTGTGGATCTTGTGGGTATGAACT |
|  | Rev | TTACCGTACTTTGATCCGATTGTC |
| LRR [AT3G43740] | Fwd | AATTCGTCTAACGGAAGCAAACTC |
|  | Rev | AACAACATTGTCTGGATCTGATAAGC |
