# Supplementary material for: Wheat Brassinosteroid-Insensitive1 (TaBRI1) Interacts with Members of TaSERK Gene Family and Cause Early Flowering and Seed Yield Enhancement in Arabidopsis
Source: PLoS One. 2016 Jun 20;11(6):e0153273. doi: 10.1371/journal.pone.0153273 (PMC4913921; doi:10.1371/journal.pone.0153273)
Supplement: S2 Table — (DOCX) [file pone.0153273.s003.docx]

**S2 Table. List of upregulated genes obtained from microarray analysis**
